# Theoretical simulation of the infrared signature of mechanically stressed polymer solids

**DOI:** 10.3762/bjoc.13.165

**Published:** 2017-08-17

**Authors:** Matthew S Sammon, Milan Ončák, Martin K Beyer

**Affiliations:** 1 Institut für Ionenphysik und Angewandte Physik, Universität Innsbruck, Technikerstraße 25, 6020 Innsbruck, Austria

**Keywords:** density functional theory, infrared spectroscopy, mechanical stress, polyamide, polyester

## Abstract

Mechanical stress leads to deformation of strands in polymer solids, including elongation of covalent bonds and widening of bond angles, which changes the infrared spectrum. Here, the infrared spectrum of solid polymer samples exposed to mechanical stress is simulated by density functional theory calculations. Mechanical stress is described with the external force explicitly included (EFEI) method. The uneven distribution of the external stress on individual polymer strands is accounted for by a convolution of simulated spectra with a realistic force distribution. *N*-Propylpropanamide and propyl propanoate are chosen as model molecules for polyamide and polyester, respectively. The effect of a specific force on the polymer backbone is a redshift of vibrational modes involving the C–N and C–O bonds in the backbone, while the free C–O stretching mode perpendicular to the backbone is largely unaffected. The convolution with a realistic force distribution shows that the dominant effect on the strongest infrared bands is not a shift of the peak position, but rather peak broadening and a characteristic change in the relative intensities of the strongest bands, which may serve for the identification and quantification of mechanical stress in polymer solids.

## Introduction

Mechanical stress on polymer solids leads to conformational changes, bond elongation and widening of bond angles on the molecular level [1–4]. If the local force on an individual polymer strand reaches values in the range of nN, rupture of covalent bonds becomes possible, leading to irreversible changes and the destruction of the molecule [5–8]. In addition, new minima on the potential energy surface (PES) might become available through relaxation due to the applied force [9]. Covalent bond rupture plays an important role in stress-induced aging of polymeric materials [1,10]. On the other hand, elegant routes have been established to harness this effect for the design of self-healing and stress-responsive materials [11–15]. The influence of an external force on the molecular structure of a polymer can be followed by recording infrared spectra [16–29]. External force modifies the force constants of vibrational modes [30]. Since structural deformation changes the charge distribution in the molecule, the transition dipole moment and thus the infrared intensity is influenced as well [30], resulting in the observed force-dependent shift of the infrared bands and changes in the intensity.

Computational chemistry has proven to be an indispensable tool in the analysis of mechanochemical phenomena of organic molecules, polymers and mechanophores [5–6,31–74]. A variety of theoretical approaches have been developed to model external force using methods of quantum chemistry [9,75–76], including constrained geometries simulate external force (COGEF) [4], external force is explicitly included (EFEI) [61,77] and force modified potential energy surface (FMPES) [45]. Within the EFEI method, force is applied along the direction defined by two atoms in the molecule, which modifies the potential energy surface, closely resembling FMPES. With EFEI, standard quantum chemical tasks like geometry optimization, reaction path following [54,56,68] and frequency calculations can be performed with minor modifications of standard packages. UV–vis, Raman and IR spectra of small model molecules exposed to mechanical stress have been calculated in this way [30,78–79]. Calculated vibrational frequencies have been employed in the theoretical modeling of force-dependent silyl ester hydrolysis rates [33]. The judgement of energy distribution (JEDI) tool developed by Stauch and Dreuw relies on the Hessian matrix in redundant internal coordinates under the influence of an external mechanical force [75,80].

So far, most studies on infrared spectroscopy of stressed polymers focused on polypropylene [30]. Lacking a pronounced infrared chromophore, however, the spectrum is relatively complicated, especially since a large number of C–H stretching, bending and wagging modes are more or less strongly coupled [30]. In the present study, we therefore focus on molecules with strong infrared chromophores, such as C–N and C–O groups. In particular, we choose *N*-propylpropanamide and propyl propanoate as model molecules for polyamide and polyester, respectively. To facilitate a comparison with future experimental studies, we convolute simulated infrared spectra with the exponential force distribution recently derived by Adhikari and Makarov for elastomeric polymer networks [81].

## Results and Discussion

### Amide

**Scheme 1:**
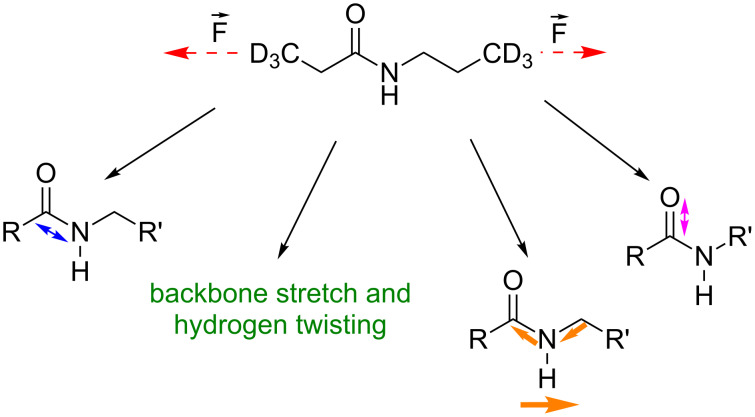
*N*-Propylpropanamide and characteristic infrared active vibrational modes. Modes are in order of lowest (left) to highest (right) vibrational frequency. Animations of the vibrations are given in Supporting Information File 1.

We investigate force-induced changes on an *N*-propylpropanamide molecule. By applying an external force to the terminal C-atoms of *N*-propylpropanamide, Scheme 1, the calculated distance between them increases from 7.43 to 8.33 Å when the force is increased from 0 nN to 4 nN in steps of 0.1 nN. Due to the vector property of the applied force, the change of the vibrational modes with increasing force depends on the orientation of the normal mode displacement of each atom relative to the force vector.

**Figure 1:**
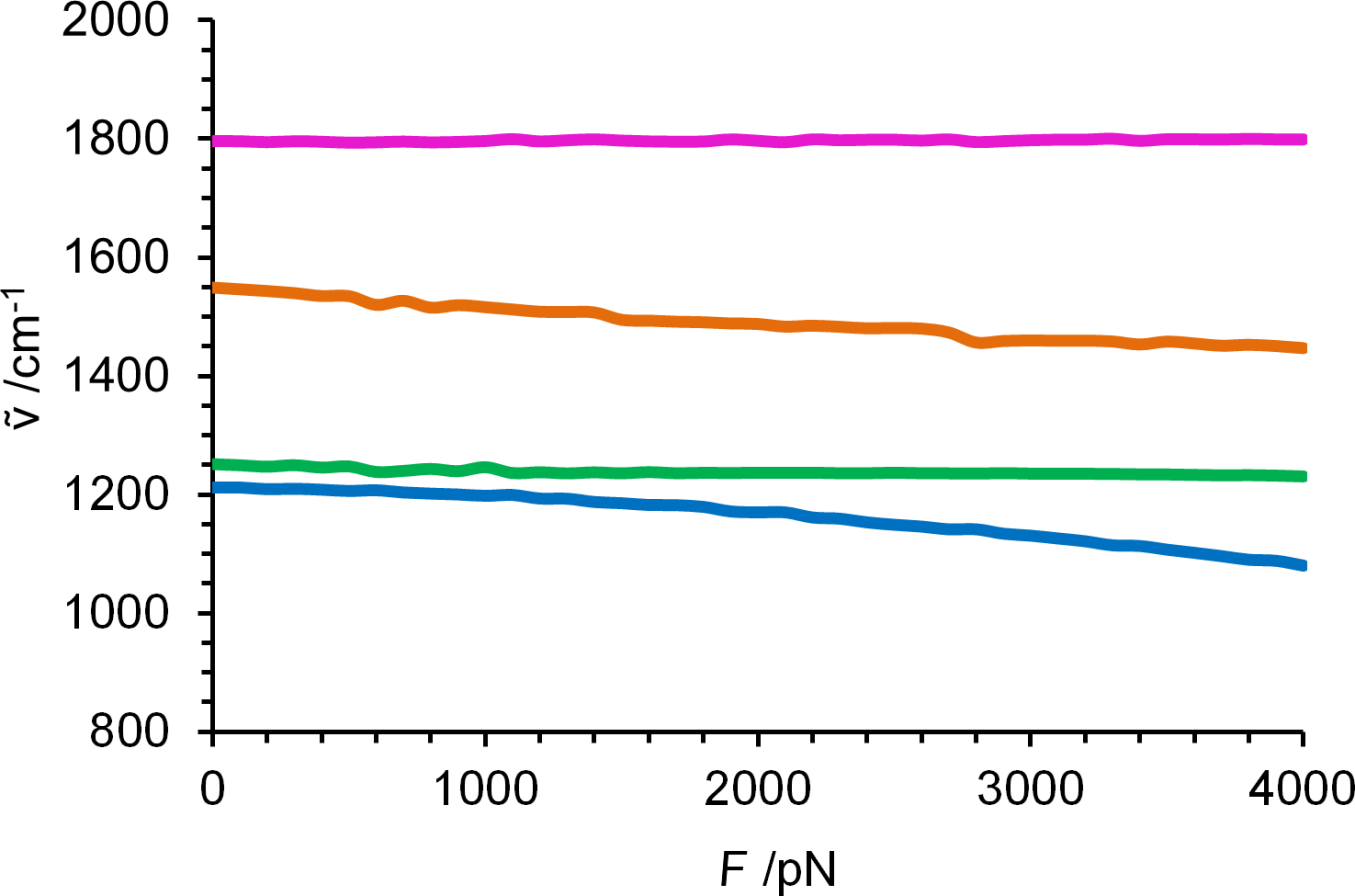
Force dependence of the modes shown in Scheme 1 in the fingerprint region from 800 to 2000 cm^−1^. C–N stretch of the amide bond (blue); backbone stretch combined with C–H_2_ twisting (green); N–H wagging (orange); free C–O stretch (purple).

To illustrate the molecular origin of the changes in the calculated infrared spectrum due to force, the four characteristic vibrational modes illustrated in Scheme 1 were chosen. Figure 1 shows their vibrational frequency as a function of force. The C–N stretching mode in the backbone, in which the carbon atom from the amide bond is involved, shows a significant redshift when external force is applied, shifting from 1212 cm^−1^ at 0 nN to 1080 cm^−1^ at 4 nN. This is explained by the elongation of the molecule, which weakens the bond and reduces the force constant. Since the influence of the external force is most pronounced in the backbone, the C–N stretching mode shows the strongest shift among the four characteristic vibrational modes. A weak backbone stretch coupled with twisting of the CH_2_ groups occurs between 1251 cm^−1^ and 1230 cm^−1^ and will be discussed exemplarily for the force influence on weaker C–H vibrations. It changes monotonically over the entire force range and experiences a moderate shift of −21 cm^−1^, compared to −132 cm^−1^ for the C–N stretching vibration. With increasing external force the coupling with neighboring CH_2_ groups decreases significantly (see animations in Supporting Information File 1). A second C–N stretching mode in the backbone, which is accompanied by an N–H wagging mode, again exhibits a strong negative force dependence, with the frequency shifting from 1549 to 1447 cm^−1^. The dominant motion of the free C–O stretching mode at 1793–1800 cm^−1^ is perpendicular to the external force, which explains the absence of a significant shift.

As shown before, vibrational modes involving the backbone exhibit a strong force dependence [30]. What is surprising, however, is the almost complete insensitivity of the free C–O stretch. One might expect that the deformation of the amide bond in the backbone changes the electron distribution, and that the weakening of the C–N bond in the backbone is compensated by a strengthening of the free C–O bond. This is obviously not the case as the bond seems to be completely unaffected by the external force, which is in line with a negligible change of the C–O bond length, from 1.22 to 1.21 Å.

**Figure 2:**
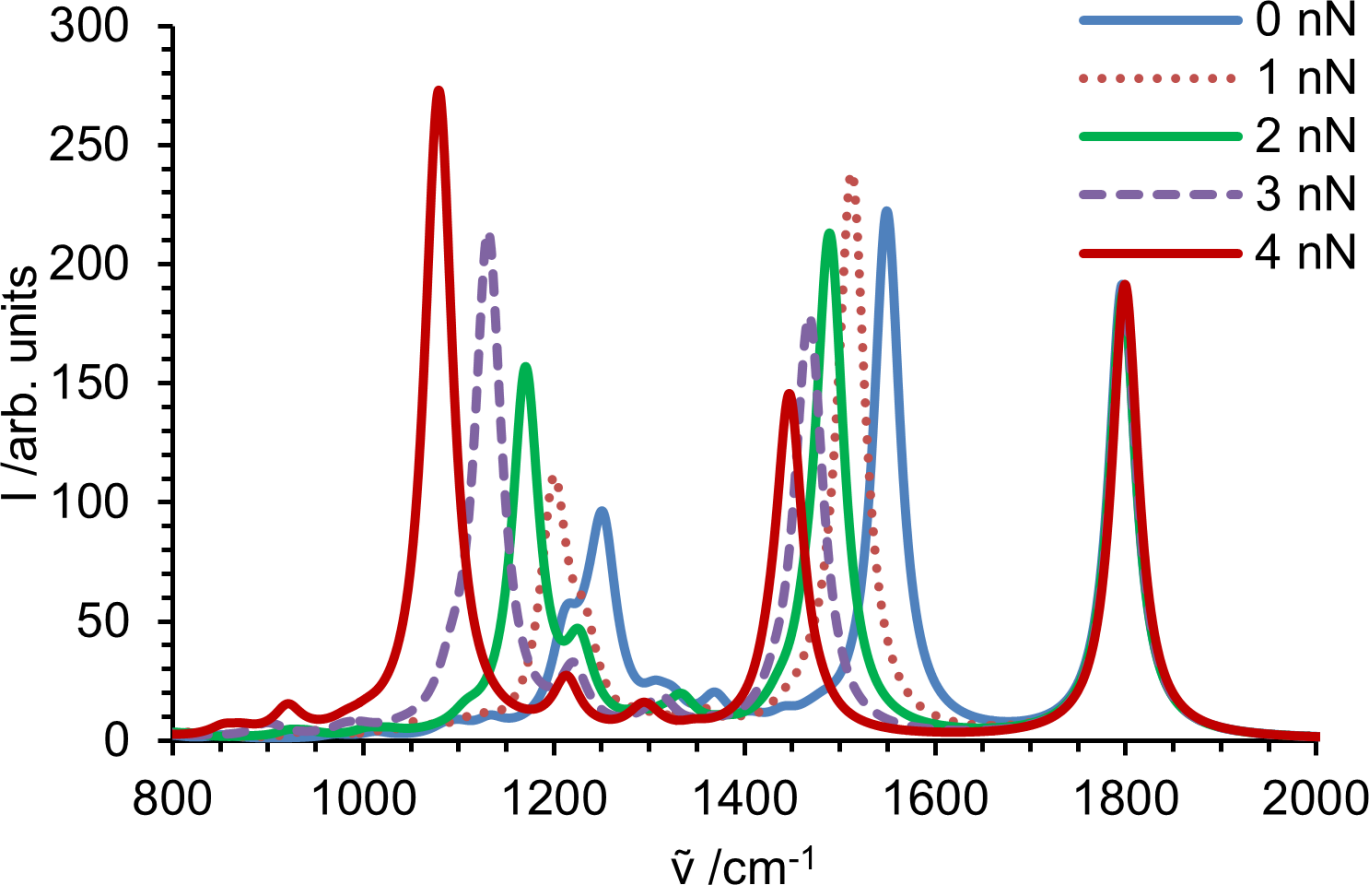
Intensities in fingerprint region of the infrared spectrum obtained for *N*-propylpropanamide. Spectral lines are broadened with a Lorentzian (34 cm^−1^ at FWHM) and summed up, yielding the spectrum of a stretched molecule.

The calculated spectra in the fingerprint area are given in Figure 2. Since the modes show different force dependences, spectral overlap and coupling of different modes can significantly influence the peak intensities. This leads to the significant change of the overall shape of the spectrum. While an external force does not influence the C–O stretching vibration, IR bands mainly attributed to modes including backbone vibrations show a considerable change in intensities. The intensity of the C–N stretching vibration in the range of 1000–1220 cm^−1^ continuously increases with increasing force due to a stronger dipole moment change resulting from interatomic bond elongation in the backbone.

**Figure 3:**
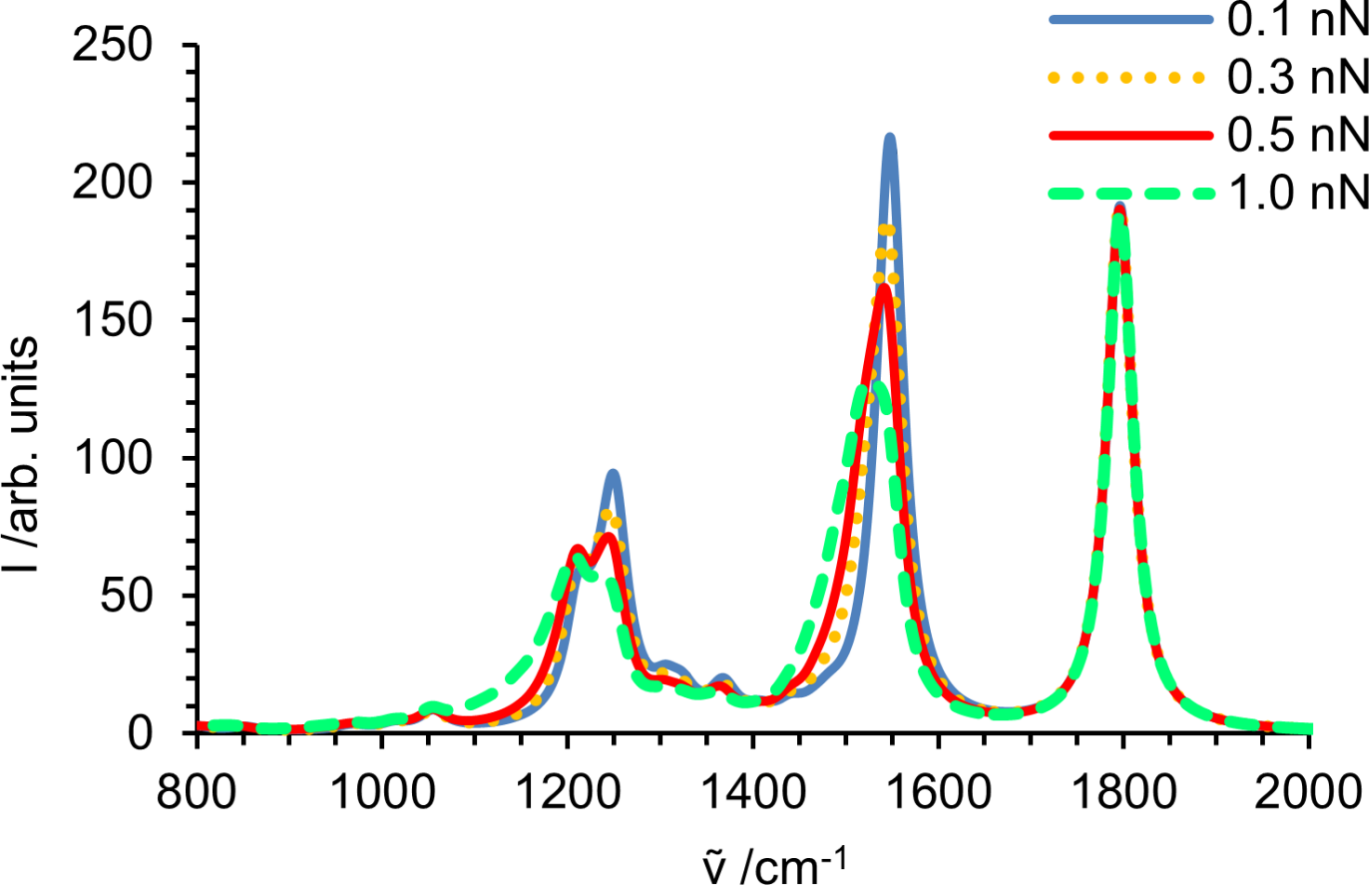
Fingerprint region of a simulated spectrum of an *N*-propylpropanamide solid sample at 0.1, 0.3, 0.5 and 1.0 nN mean force per polymer strand.

However, the spectra of molecules exposed to a specific force, shown in Figure 2, cannot be compared with experimental data. In a polymer solid, the individual polymer strands experience a broad distribution of forces. Adhikari and Makarov have recently shown for elastomeric polymer networks that an exponential distribution is an excellent approximation [81]. It is straightforward to calculate spectra as a convolution of the spectra at specific forces with the exponential force distribution. The result of this convolution is displayed in Figure 3 for mean forces of 0.1 to 1 nN. Since the most probable force is close to zero, the peak position does not change dramatically. The bands originating from strongly red shifting modes are only slightly red shifted, but significantly broadened. Moreover, the broadening leads to a significantly decreased peak height of the band around 1550 cm^−1^. The band around 1250 cm^−1^ is composed of several vibrational modes, and their different force dependence leads to seemingly erratic changes in peak shape. Interestingly, the change in the peak shape with increasing force resembles the experimentally observed difference between bulk and surface spectra reported by Vettegren and co-workers [27].

Since the peak of the C–O stretching vibration does not change with force, the relative intensity of the two strong bands around 1550 cm^−1^ and 1800 cm^−1^ may actually serve as a direct measurement of the mechanical stress experienced locally in a polymer solid.

### Ester

Another technically relevant polymer is polyester, for which propyl pronanoate was chosen as model molecule. According to our EFEI geometry optimizations, the distance between terminal C-atoms in propyl propanoate increases from 7.42 to 8.19 Å when an external force of 4 nN is applied. This elongation of 0.77 Å is significantly smaller than for the previously discussed *N*-propylpropanamide with 0.90 Å.

**Scheme 2:**
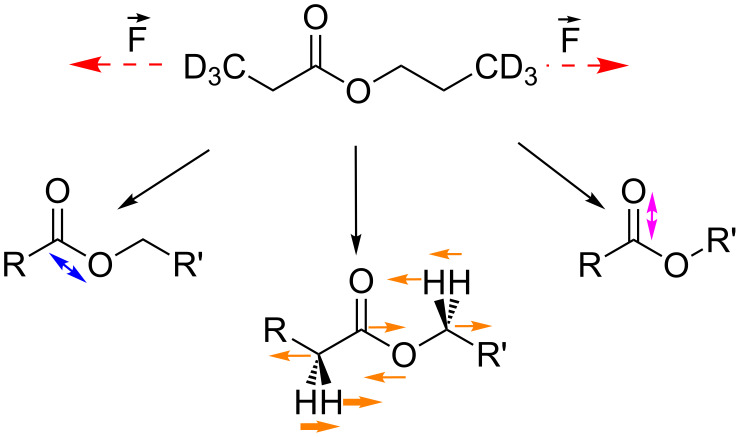
Propyl propanoate and characteristic infrared active vibrational modes. Modes are in order of lowest (left) to highest (right) vibrational frequency. For the second mode bold arrows describe the main vibrational mode and plain arrows coupled modes. Animations of the vibrations are given in Supporting Information File 1.

**Figure 4:**
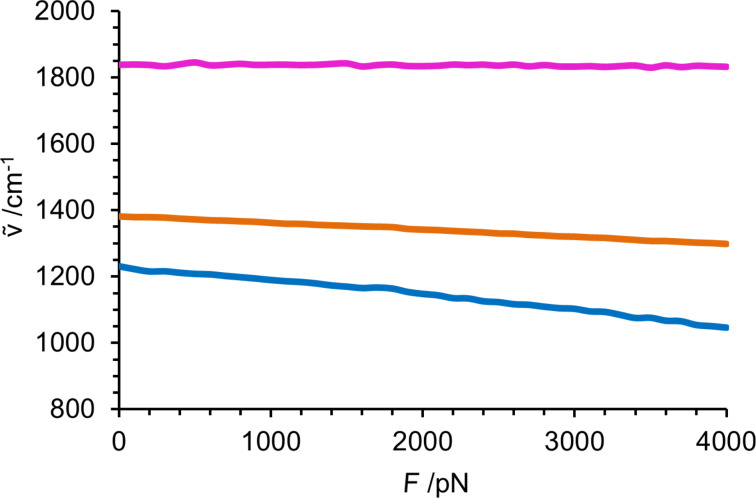
Force dependence of the modes shown in Scheme 2 in the fingerprint region from 800 to 2000 cm^−1^. C–O backbone stretch of the ester bond (blue); backbone stretch combined with C–H_2_ wagging (orange); free C-O stretch (purple).

For propyl propanoate, the three representative vibrational modes shown in Scheme 2 were selected in the fingerprint region and followed over the calculated force range of 0–4 nN, Figure 4. The C–O backbone-stretching mode exhibits the strongest negative force dependence, shifting from 1231 to 1046 cm^−1^, due to its strong alignment with the external force vector. If no force is applied, a CH_2_ wagging mode next to the ester group, combined with a stretching vibration in the backbone (orange) is present at 1381 cm^−1^. Upon increasing the external force to 4 nN, it shifts to 1298 cm^−1^. Again, the vibrational modes involving motion of atoms along the backbone experience a strong redshift. The C–O stretching vibration (pink) perpendicular to the applied force occurs at slightly higher wavenumbers than for the amide bond, in the range of 1830–1846 cm^−1^. It is basically independent of the force applied to the molecule.

**Figure 5:**
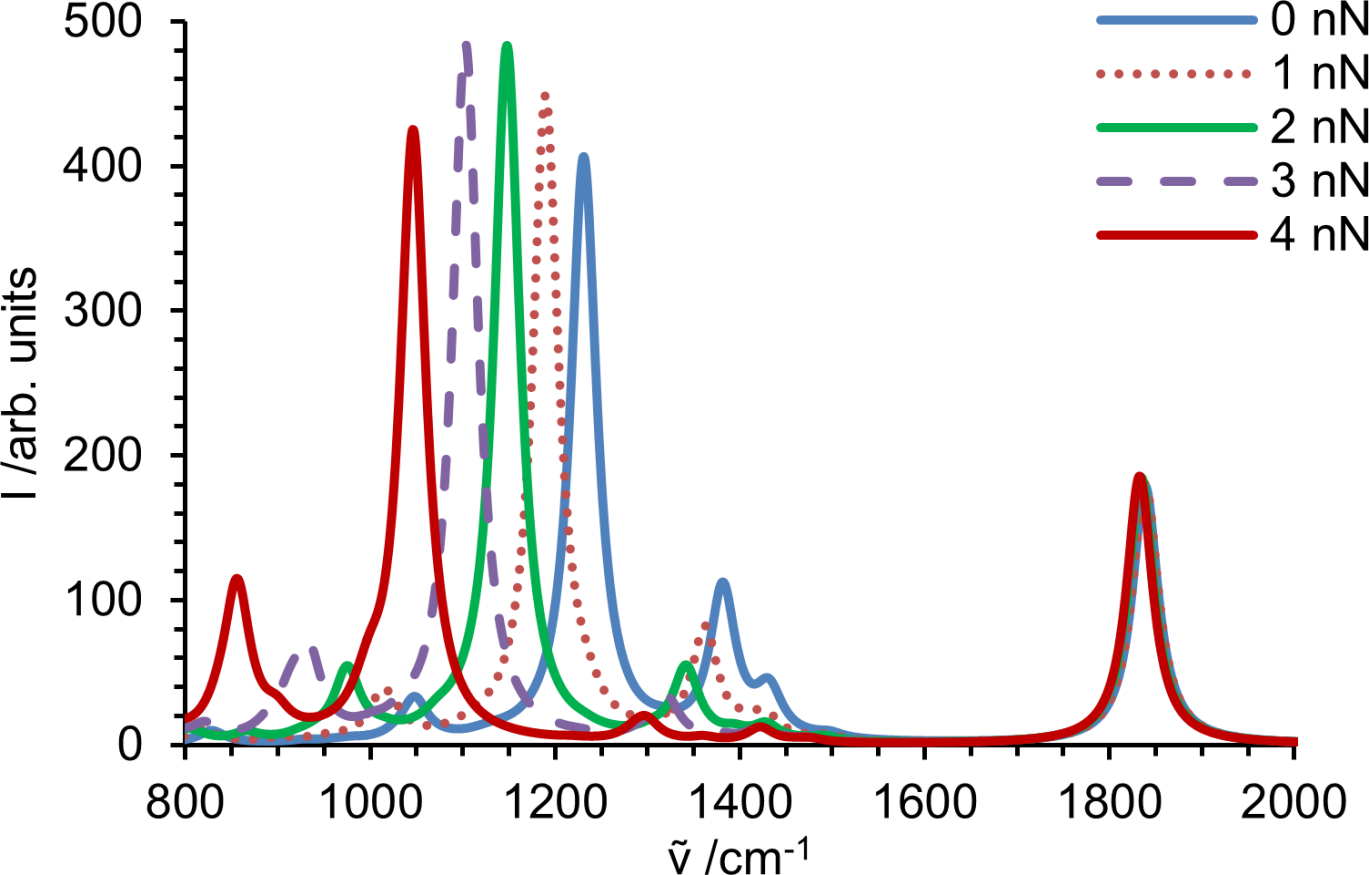
Intensities in fingerprint region of the infrared spectrum obtained for propyl propanoate. Spectral lines are broadened with a Lorentzian (34 cm^−1^ at FWHM) and summed up, yielding the spectrum of a stretched molecule.

Simulated spectra in the fingerprint region of individual molecules exposed to a specific force are presented in Figure 5. As discussed above for *N*-propylpropanamide, the intensity of the stretching vibration of the C–O double bond of propyl propanoate is not affected by external force applied at the terminal C-atoms. Modes containing vibrations along the backbone, however, experience significant changes in intensity. The wagging mode of hydrogen adjacent to the ester bond decreases in intensity and almost vanishes at 4 nN. For the C–O-stretching mode in the backbone the intensity first increases similar to the C–N vibration in the amide, reaches a maximum around 2.2 nN and then decreases again. Peaks propagating from 1047 cm^−1^ at 0 nN to 858 cm^−1^ at 4 nN result from numerous overlapping C–H and backbone vibrations. Due to their complexity, as described in detail before for polypropylene [30], as well as the lower intensity compared to vibrations from strongly IR active functional groups, they do not seem to be a valuable reference for force-dependent evaluation of the resulting spectra. Moreover, the complex interplay of different modes generating these broad absorptions may be strongly affected by the limited length of the model molecule, while the behavior of the lines originating from the ester moiety should be robust.

**Figure 6:**
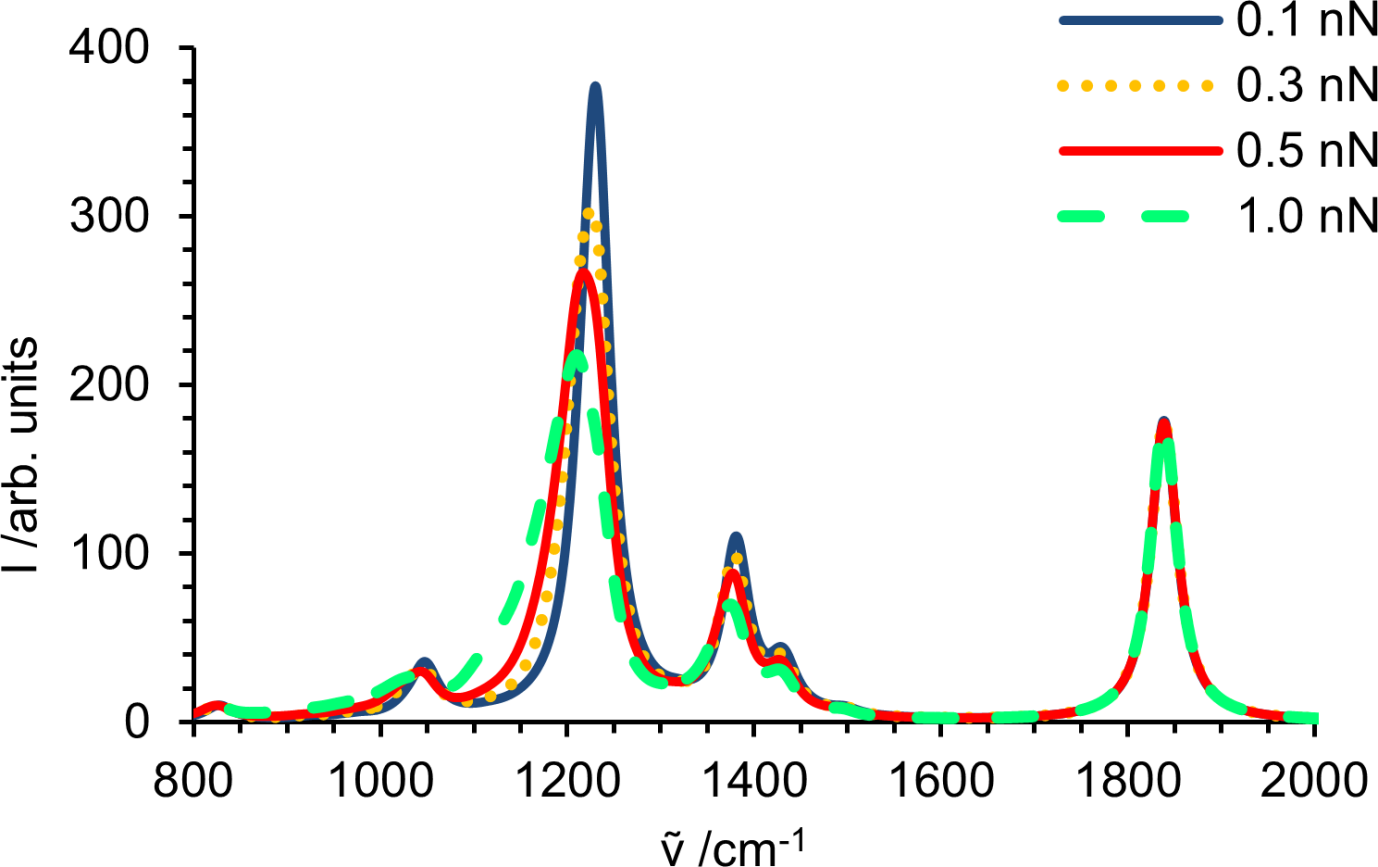
Fingerprint region of a simulated spectrum of a propyl propanoate solid sample at 0.1, 0.3, 0.5 and 1.0 nN mean force per polymer strand.

The weighted spectra, obtained again by convolution with an exponential force distribution [81], are given in Figure 6. As for the amide, the bands dominated by backbone vibrations show a significant decrease in intensity accompanied by a broadening towards smaller wavenumbers, while the free C–O stretching vibration remains unaffected.

## Conclusion

Experimental studies on the IR spectra of mechanically stressed polymers mostly focused on possible shifts in the peak position of bands around 1000 cm^−1^. These shifts, however, are due to a complex interplay of overlapping backbone modes involving CH_2_ groups, and are difficult to interpret. For polymers with strong IR active modes, like polyamides or polyesters, we have shown here that the peak positions of real samples do not shift strongly. This is due to the exponential force distribution used for the modeling, which means that most functional polymer strands are exposed to very small forces, but a small number of groups experiences very high forces. This results in a significant broadening combined with a small redshift of bands involving backbone vibrations. The peak broadening also leads to a decrease of the absorption maximum. In contrast, both peak position and intensity of the free C–O stretching mode are completely unaffected in both studied molecules. Thus, for comparison with experimental results we propose using the free C–O stretching mode as a reference band to quantify the influence of external force on the remaining strong infrared modes.

## Methods

DFT calculations were performed using the B3LYP functional along with Ahlrich’s SVP basis set. For computations, TURBOMOLE 7.0.2 was used [82–83] with a script implementing EFEI using numerical calculation of the second derivative after geometry optimization, as described by Pill et al. [30]. Initial optimization leads to a fairly consistent increase in the distance between pulling points, but includes structures with imaginary vibrational modes and abrupt conformational changes. Respective structures were re-calculated using the geometry obtained for the next higher force as starting structure (see Figure S3, Supporting Information File 1). All calculated structures represent local minima. No frequency scaling factor was applied. To validate the sufficiency of Ahlrich’s SVP basis set, calculations without applying external force were performed using the TZVP basis set. Furthermore, computations were carried out for 0 nN using numerical calculations as implemented in TURBOMOLE. See Supporting Information File 1 for details.

*N*-Propylpropanamide and propyl propanoate were used as model molecules for polyamides and polyesters, respectively. Since polymers consist of multiple repetition units, any vibrational modes specific to the polymer ends would be very weak. Therefore, hydrogen atoms from the terminal methyl groups were substituted with deuterium. The spectral lines originating from these CD_3_ groups were removed from the simulated spectra. To simulate the intensities of infrared bands in the fingerprint region at a given force, the remaining spectral lines were broadened using a Lorentzian with a full width at half maximum of 34 cm^−1^. To simulate spectra of polymer solids, the broadened spectra were convoluted with the probability distribution *P*(*F*) derived by Adhikari and Makarov [81], Equation 1, with the actual force acting on the polymer strand *F* and the mean force *<F>*.

[1]
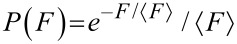


## Supporting Information

The Supporting information comprises a short description of steps taken to validate the accuracy of the methods used, the elongation of the respective model structures, animations of the vibrational modes in the fingerprint region, calculated infrared spectra and convoluted spectra over the entire frequency range. Additionally, the atomic coordinates calculated for the model molecules without external force are given.

File 1 Additional material.
