# Supplementary material for: Theoretical simulation of the infrared signature of mechanically stressed polymer solids
Source: Beilstein J Org Chem. 2017 Aug 17;13:1710–6. doi: 10.3762/bjoc.13.165 (PMC5564256; doi:10.3762/bjoc.13.165)
Supplement: File 1 — Additional material. [file Beilstein_J_Org_Chem-13-1710-s001.docx]

**Supporting Information**

**for**

**Theoretical simulation of the infrared signature of mechanically stressed polymer solids**

**Matthew S. Sammon, Milan Ončák^*^ and Martin K. Beyer^*^**

**Address: Institut für Ionenphysik und Angewandte Physik, Universität Innsbruck, Technikerstraße 25, 6020 Innsbruck, Austria**

**Email: Milan Ončák - milan.oncak@uibk.ac.at; Martin K. Beyer - martin.beyer@uibk.ac.at**

***Corresponding author**

**Additional material**

**Figure S1:** Testing the EFEI script for consistency. Calculations were performed with a script for implementing EFEI and using the B3LYP functional with Ahlrich’s SVP basis set. The correct use of the numerical method from TURBOMOLE was checked by comparing results obtained with the script at 0 nN external force with those from only using numerical calculations from TURBOMOLE, as seen in Figure S1.

**Figure S2:** We tested the sufficiency of Ahlrich’s SVP basis set by comparison with IR spectra obtained using the larger TZVP basis set (Figure S2) at 0 nN external force, showing minor changes of peak positions and intensities.

**Figure S3:** Consistency of geometry optimizations in steps of 0.1 nN. Optimization of the structures was performed using the optimized geometry of the previous force step as starting point. This worked well for most force steps, verified by the steady elongation of the backbone with increasing force as shown in Figure S3. Single outliers and structures showing imaginary vibrational modes were re-calculated using the optimized coordinates for the next higher force as starting geometry. The resulting structures had no vibrational modes with imaginary frequencies, and the backbone length increase was more consistent.

**Scheme S1:** Animations of followed vibrational modes at 0 nN and 4 nN external force.

**Figures S4 and S5:** Calculated IR spectra and weighted spectra over the entire frequency range.

**Coordinates:** The atomic coordinates of *N*-propylpropan­amide and propyl propanoate are given after geometry optimization without external force.

**B)**

**A)**

**Figure S1:** Spectra obtained after broadening spectral lines from numerical calculations for amide (N-propylpropanamide, A) and ester (propyl propanoate, B) before omitting C–D vibrations. Comparison of the spectra calculated with the EFEI script implemented in TURBOMOLE (used in this work) at 0 nN with the spectra only using TURBOMOLE with numerical second derivative calculations shows almost no differences regarding position and intensity of IR bands.

**B)**

**A)**

**Figure S2:** IR spectra obtained from calculations using Ahlrich’s SVP basis set (blue) compared to those from calculations using TZVP (red) for deuterated amide (A) and ester (B). Vibrations resulting from deuteration of terminal methyl groups were not omitted. Slight differences in position and intensities are seen for both molecules. However, the positions and intensity ratios between the bands are similar.

**B)**

**A)**

**Figure S3:** Distance d in Å between terminal C atoms of amide (blue) and ester (red) obtained from initial optimization (A) and subsequent consistency adjustment (B).

**A)**

**B)**

**

**

**Scheme S1:** Followed representative vibrational modes of *N*-propylpropanamide (A) and propyl propanoate (B), respectively. The embedded gif-files are animations of these modes at 0 nN and 4 nN external force. Double-click to watch animated gifs.

**A)**

**B)**

**Figure S4:** Spectra obtained from broadening using a Lorentzian (34 cm^−1^ at FWHM) for 0 (blue), 2 (green) and 4 nN (red) of external force for amide (A) and ester (B), from which C–D vibrations were omitted. Bands around 3000 cm^−1^ are attributed to C–H vibrations, in the amide spectrum around 3600 cm^−1^ to N–H vibrations.

**A)**

**B)**

**Figure S5:** Weighted spectra obtained from broadening using a Lorentzian (34 cm^−1^ at FWHM) for 0.1 (blue), 0.5 (green) and 1.5 nN (red) of external force for amide (A) and ester (B), from which C–D vibrations were omitted.

*Coordinates for N-propylpropanamide at 0 nN (in a.u.):*

-4.59436142564122 -2.54611000557067 2.68027033957168 c

-2.44476299514779 -2.07798176024846 4.54754010732681 c

0.08915391570457 -3.08924347402306 3.58616254532595 c

0.60086592911342 -3.18031435048877 1.33966959058884 o

1.74072180582305 -3.82212450911388 5.43686192301927 n

4.28070091414586 -4.72636084334315 4.93208439004491 c

6.31301404226697 -3.22191764253055 6.34320358627490 c

8.98245732089764 -4.21303455045270 5.85587178765859 c

-4.07168756709627 -1.82489030465388 0.80261228690500 h

-6.34452386058504 -1.60708951718772 3.30032471272405 h

-4.99126617734983 -4.57997714753929 2.47753890204833 h

-2.88133594359014 -2.87658930465811 6.42613261957688 h

-2.19396670185014 -0.02406168612007 4.83945943028431 h

4.43756582986375 -6.75213158845360 5.43371747941648 h

4.54791115352812 -4.58837313456558 2.87466437988864 h

6.17341348935809 -1.21608493842766 5.78563675567598 h

5.91003648205962 -3.28517224783831 8.39380122482863 h

10.39733294699722 -3.13230615127647 6.93467753451051 h

9.16399858896260 -6.21622186192183 6.40608132516352 h

9.48340261735017 -4.07577804179037 3.83766577567687 h

1.16129929176810 -3.73885627758092 7.25982576332086 h

*Coordinates for propyl propanoate at 0 nN (in a.u.):*

-0.15260507624445 0.39345218518094 -0.00980788546109 c

0.12027456481418 -0.17537354587409 2.80157156205730 c

1.70728727038519 0.62167026958418 -0.91334377627823 h

-1.24214735515591 2.14055759728770 -0.30665391132426 h

-1.12408011709458 -1.16007215569164 -0.99382205471936 h

-1.73605716710272 -0.32722599814726 3.74243385118832 h

1.53467248595729 -2.61176394361643 3.32619102277457 c

1.11276687316436 1.35681183434371 3.81123784079033 h

1.77892986593422 -3.01415235694633 5.82847499423587 o

2.34490653467424 -4.03980607778390 1.74265646474866 o

3.05483642148826 -5.28451197987161 6.58774285954532 c

3.15562726166275 -5.34274325195005 9.45695602574059 c

2.03350301407178 -6.93217712446765 5.81834448129408 h

4.96219905312702 -5.31509202464079 5.74564333453529 h

4.45328943448642 -7.72076578691250 10.46396052979291 c

4.15043433515186 -3.63538889223077 10.12571418200315 h

1.21009149953246 -5.21340156149152 10.19721067145989 h

4.50445996050130 -7.72153832323362 12.54391749569483 h

3.45864151652698 -9.45013357986345 9.85982215838322 h

6.41967130971275 -7.86308956966323 9.78818045151903 h
